# Pneumococcal Phasevarions Control Multiple Virulence Traits, Including Vaccine Candidate Expression

**DOI:** 10.1128/spectrum.00916-22

**Published:** 2022-05-10

**Authors:** Zachary N. Phillips, Claudia Trappetti, Annelies Van Den Bergh, Gael Martin, Ainslie Calcutt, Victoria Ozberk, Patrice Guillon, Manisha Pandey, Mark von Itzstein, W. Edward Swords, James C. Paton, Michael P. Jennings, John M. Atack

**Affiliations:** a Institute for Glycomics, Griffith University, Queensland, Australia; b Research Centre for Infectious Diseases, Department of Molecular and Biomedical Science, University of Adelaide, Adelaide, Australia; c Department of Medicine, Division of Pulmonary, Allergy, and Critical Care Medicine, University of Alabama at Birmingham, Birmingham, Alabama, USA; d School of Environment and Science, Griffith University, Southport, Australia; Emory University School of Medicine

**Keywords:** phasevarion, phase variation, *Streptococcus pneumoniae*, vaccine, epigenetic regulation, pneumococcus

## Abstract

Streptococcus pneumoniae is the most common cause of bacterial illness worldwide. Current vaccines based on the polysaccharide capsule are only effective against a limited number of the >100 capsular serotypes. A universal vaccine based on conserved protein antigens requires a thorough understanding of gene expression in S. pneumoniae. All S. pneumoniae strains encode the SpnIII Restriction-Modification system. This system contains a phase-variable methyltransferase that switches specificity, and controls expression of multiple genes—a phasevarion. We examined the role of this phasevarion during pneumococcal pathobiology, and determined if phase variation resulted in differences in expression of currently investigated conserved protein antigens. Using locked strains that express a single methyltransferase specificity, we found differences in clinically relevant traits, including survival in blood, and adherence to and invasion of human cells. We also observed differences in expression of numerous proteinaceous vaccine candidates, which complicates selection of antigens for inclusion in a universal protein-based pneumococcal vaccine. This study will inform vaccine design against S. pneumoniae by ensuring only stably expressed candidates are included in a rationally designed vaccine.

**IMPORTANCE** S. pneumoniae is the world’s foremost bacterial pathogen. S. pneumoniae encodes a phasevarion (phase-variable regulon), that results in differential expression of multiple genes. Previous work demonstrated that the pneumococcal SpnIII phasevarion switches between six different expression states, generating six unique phenotypic variants in a pneumococcal population. Here, we show that this phasevarion generates multiple phenotypic differences relevant to pathobiology. Importantly, expression of conserved protein antigens varies with phasevarion switching. As capsule expression, a major pneumococcal virulence factor, is also controlled by the phasevarion, our work will inform the selection of the best candidates to include in a rationally designed, universal pneumococcal vaccine.

## INTRODUCTION

Streptococcus pneumoniae, the pneumococcus, is a human-adapted bacterial pathogen of global importance (1). S. pneumoniae commonly colonizes the nasopharynx asymptomatically in both children and adults (2). Colonization may develop into diseases of the respiratory tract, and lead to life-threatening meningitis and sepsis (invasive pneumococcal disease; IPD) (1). S. pneumoniae is the predominant cause of middle ear infection (otitis media; OM), the most common bacterial infection in children (>350 million cases annually), and the leading cause of childhood antibiotic prescription and visits to health care providers in the developed world (3, 4). S. pneumoniae is also one of the most common bacterial pathogens implicated in exacerbations of chronic obstructive pulmonary disease (COPD) (5–7), although certain studies have demonstrated that the role of S. pneumoniae is COPD is not clear (8). COPD affects >380 million people globally, costing $1 billion in health care costs annually in Australia alone (9). In 2015, S. pneumoniae infection was responsible for approximately 1.5 million deaths from pneumonia alone, including 650,000 children aged <5 years (10). The prevalence, morbidity, and mortality, associated with S. pneumoniae mean it is extremely important to understand the pathobiology of this organism to develop better treatments and vaccines. Current vaccines against S. pneumoniae based on the pneumococcal capsule (conjugate vaccine PCV-13, and purified polysaccharide vaccine PPSV-23), only target a limited number of the >100 capsular serotypes (1, 11), and are only moderately effective at preventing OM (12). A universal pneumococcal vaccine based on conserved protein antigens could potentially provide complete protection against all strains.

**FIG 1:**
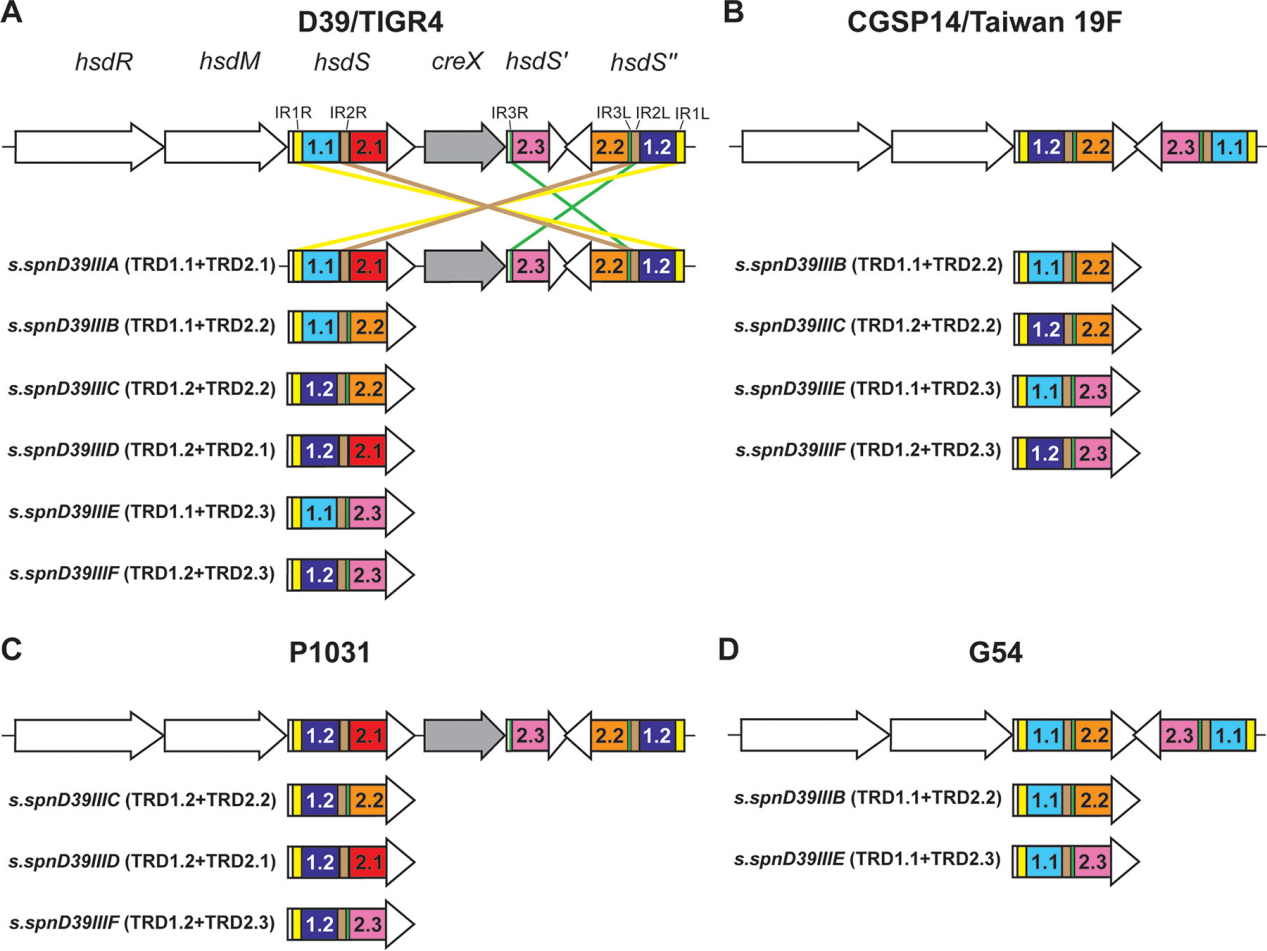
Different major forms of the SpnIII system in surveyed genomes. The SpnIII system was examined in NCBI fully annotated S. pneumoniae genomes. (A) The “full” six-way switch as first described in strains D39 and TIGR4. (B) A simpler four-way switch as seen in strain CGSP14 and Taiwan 19F. (C) A three-way switch seen in strain P1031. (D) A two-way switch seen in strain G54. Further details can be found in Table S1. All TRD fragments of the same name (e.g., 1.1) are identical.

Restriction Modification (R-M) systems are almost ubiquitous in bacteria, where they were originally characterized as a defense against foreign DNA, typically from infection by bacteriophage (13). In these systems, the restriction (R) enzyme cleaves foreign DNA at a particular DNA target sequence, while the cognate methyltransferase (modification; M) protects host DNA by methylating this same target sequence on host DNA (14). In addition, many methyltransferases associated with R-M systems have a role in gene regulation. In a number of bacterial pathogens, the methyltransferase is phase-variable. Phase variation is the rapid and reversible switching of gene expression, and is usually associated with bacterial surface features (15). Phase-variable expression of DNA methyltransferases leads to different methylation patterns in a bacterial population, dependent on the phase-variable state of the methyltransferase in each individual bacterium in the population. These methylation differences influence expression of multiple genes epigenetically, in systems called phasevarions (phase-variable regulons) (16). Phasevarions have been characterized in many bacterial pathogens such as *Neisseria* spp. (17), Moraxella catarrhalis (18), Haemophilus influenzae (19, 20), and Streptococcus suis (21). In every case, phasevarions regulate genes involved in pathobiology, and frequently genes encoding vaccine candidates. S. pneumoniae encodes a phase-variable type I R-M system, the SpnD39III phasevarion (22), herein abbreviated to SpnIII. Type I R-M systems encode restriction (*hsdR*), modification (*hsdM*), and specificity (*hsdS*) subunits. The *hsdR* gene encodes the restriction endonuclease, while the *hsdM* gene encodes the cognate methyltransferase. The *hsdS* gene encodes a specificity subunit, HsdS, which determines the sequence cleaved or methylated by the HsdR and HsdM, respectively (14). An R_2_M_2_S pentamer forms the active restriction enzyme, while an M_2_S trimer forms an active, stand-alone methyltransferase (14). Each HsdS is composed of two target recognition domains (TRDs), with each TRD contributing half to the overall specificity of the complex (16). A version of the SpnIII system is present in every publicly available genome of S. pneumoniae (>200 strains) (22). The *hsdR* and *hsdM* genes of the SpnIII system are highly conserved between strains, and the methyltransferase of the SpnIII system is always expressed. The SpnIII locus encodes multiple, variable *hsdS* loci, and a recombinase. Homologous recombination between the variable *hsdS* loci (expressed *hsdS*, and downstream silent *hsdS′* and *hsdS′′* genes) leads to multiple HsdS protein variants being expressed in a population. In strain D39 (22) and TIGR4 (23), this leads to six distinct DNA methyltransferase specificities being expressed in a pneumococcal population—SpnIII alleles A to F. These six different methyltransferase specificities result in six different gene expression profiles, resulting in six distinct phasevarions in a pneumococcal population (Fig. 1A). Studies of the phenotypic effects of the SpnIII phasevarion in S. pneumoniae strains D39 and TIGR4 have found differential regulation of virulence determinants such as opacity, capsule, and biofilm formation commensurate with phase variation between methyltransferase specificities A to F (22–24). The presence of the SpnIII phasevarion complicates the identification of conserved antigens, as the full impact of this system on pneumococcal gene regulation and pathobiology has not been investigated. Understanding the influence of the SpnIII phasevarion on gross pneumococcal phenotype, and specific effects regarding expression of conserved protein antigens, is essential to designing successful treatments and vaccines.

## RESULTS

### Examination of fully annotated pneumococcal genomes reveals not all strains encode a six-way switch.

The SpnIII system has been described as a six-way switch containing five characterized TRDs and a CreX recombinase (22). All TRD fragments of the same name (e.g., 1.1) are essentially identical, as previously shown by Oliver et al. (25). We surveyed fully annotated S. pneumoniae genomes (82 strains total) in NCBI GenBank for the presence and conservation of the “full” SpnIII system described previously (22). Fig. 1 illustrates examples of the diversity of the SpnIII system. Fig. 1A is the full six-way switch as seen in strains D39 and TIGR4 (22, 23), and is found in 68.3% of strains examined (56 strains). 8.5% of strains (seven strains), such as Taiwan 19F, have lost a TRD, and consequently this system only results in four unique HsdS proteins; a four-way switch producing alleles B, C, E, and F (Fig. 1B). 18.3% of strains (15 strains) encode the same TRD more than once, such as strain P1031, that encodes TRD 1.2 in both the *hsdS* and *hsdS*′′ loci (Fig. 1C), producing a three-way switch, in this case, between alleles C, D, and F. There are then combinations of these two factors—loss of TRDs and a duplication of one TRD, resulting in the two-way switch as seen in strain G54, which can only produce alleles B and E (Fig. 1D). This arrangement was observed in 2.4% of surveyed genomes (two strains). Another aspect we observed was the loss of the gene encoding the CreX recombinase (*creX*) in multiple strains (Fig. 1B and D). The loss of this recombinase has been observed to decrease the rate of switching, but not completely prevent it (26). 6.1% of strains had a SpnIII system which could not be placed into the above-described groups due to the loss or gain of identifiable features (five strains). For example, strain 4041STDY6836167 (a serotype 9V, sequence type [ST]156 isolate) contained a SpnIII system with seven *hsdS* regions, and strain HU-OH contained a SpnIII system with only two *hsdS* regions, one of which had <50% identity to known *hsdS* sequences (Table S1A). 6.1% of genomes were also found to have two SpnIII systems. The effects of these uncharacterized TRDs, and significance of not encoding a functional SpnIII phasevarion are outside the scope of this work. All data are presented in Tables S1A and 1B.

### Expression of different HsdS alleles of the full SpnIII phasevarion result in diverse, clinically relevant phenotypes.

**FIG 2:**
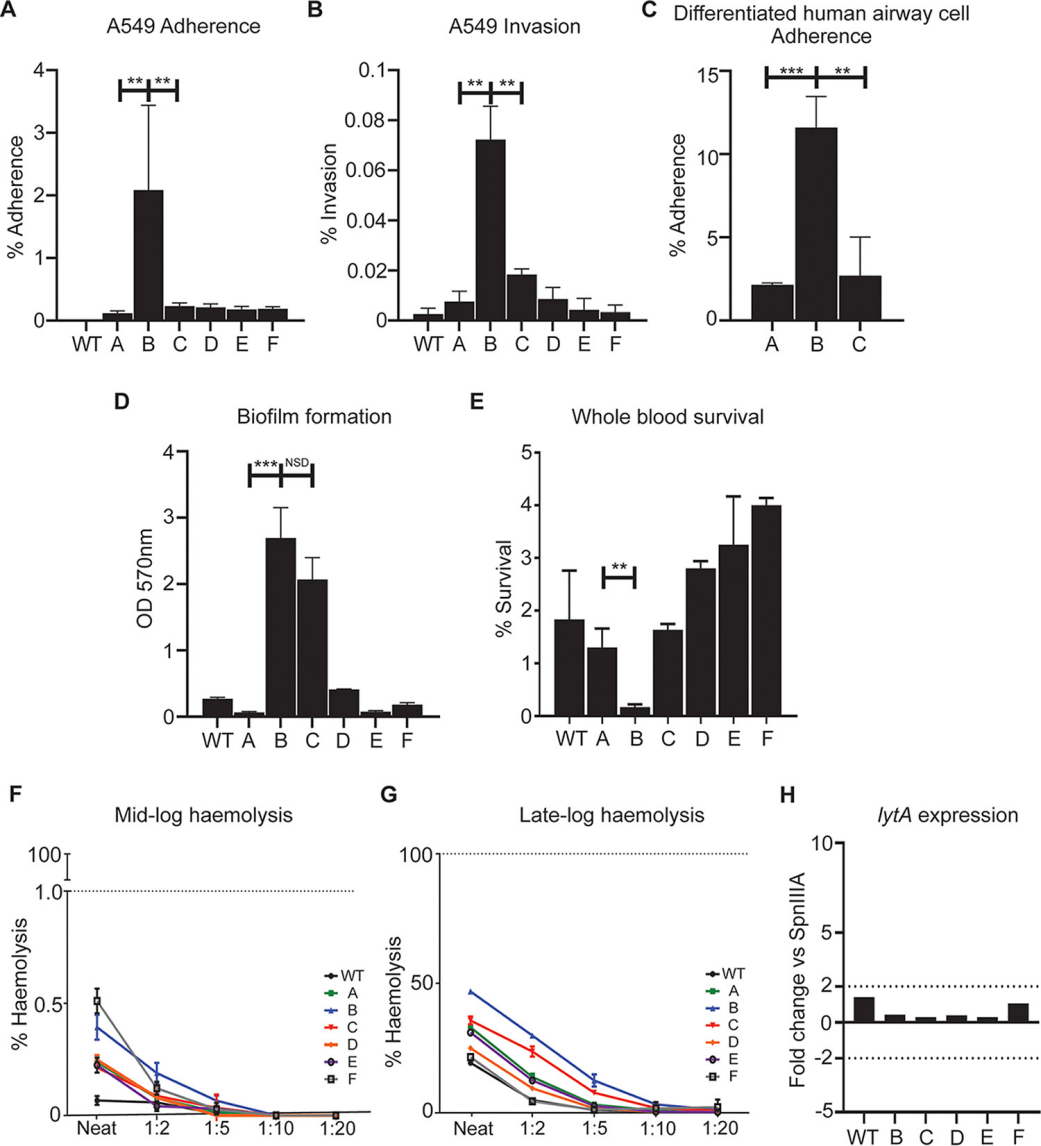
Clinically relevant traits of the SpnIII system using strain D39. (A) Adherence of strains expressing SpnIII alleles to A549 cells after 1 h. Values represent percent (%) of inoculum that was adherent. (B) Invasion assay measuring invasive ability of strains expressing SpnIII alleles after 1 h of incubation, then 45 min of antibiotic treatment. Values represent percent of inoculum that invaded A549 cells. SpnIII B had significantly better invasive ability compared with all other alleles. (C) Adherence to differentiated human airway epithelial cells after 1 h. Values represent percent of inoculum that adhered to cells. SpnIII B is significantly more adherent versus A and C. (D) Static biofilm formation was assessed after 24 h via crystal violet absorbance assay. SpnIII B and C form a significantly denser static biofilm compared to strains expressing other alleles. (E) Ability of SpnIII alleles A to F assessed for their survival in whole human blood (group O+) for 1.5 h. SpnIII B showed attenuated (~0%) survival, whereas SpnIII E and F showed greatest mean survival (~3% to 4%). Ability of SpnIII A to F alleles (strain D39) to lyse Human erythrocytes (group O+) measured at (F) mid log (OD O.5) and (G) late log (OD 1.0) growth phases after 1 h. Cells expressing SpnIII allele B had the highest consistent hemolytic ability compared with other locked alleles and WT S. pneumoniae D39. Evaluating *lytA* expression in late log samples (H) showed that no significant difference between locked SpnIII alleles A to F or WT D39. This indicates differences in hemolytic ability seen in (F) and (G) are independent of *lytA* expression. MOI 100:1. See Fig. S1 for challenge ratios. *, *P < *0.05; **, *P < *0.01; ***, *P < *0.001.

To compare clinically relevant phenotypes of the SpnIII phasevarion in strain D39, we used our six “locked” D39 mutants expressing only one of the SpnIII alleles (22). Adherence to (Fig. 2A) and invasion of (Fig. 2B) human lung A549 cells was investigated. Adherence was also examined using differentiated human nasal airway epithelial (HNAE) cells (Fig. 2C). These cells are polarized, and produce mucous. Expression of SpnIII allele B resulted in significantly more adherence to and invasion of human airway cells compared with both WT and the five other locked strains. Similar results were found using the polarized airway cell model—expression of SpnIII allele B resulted in significantly more adherence than SpnIII allele A and SpnIII allele C. When studying biofilm formation, a trait particularly important in middle ear infections, strain D39 expressing either SpnIII allele B or C showed significantly greater biofilm mass than either WT or the four other locked strains (A, D, E, F) (Fig. 2D). Strains expressing alleles A and E showed the lowest level of biofilm formation under the conditions tested.

### SpnIII phase variation affects survival in blood and hemolytic activity.

All locked strains and WT exhibited 1% to 5% survival in human blood except the strain expressing SpnIII allele B, which was unable to survive at all in whole human blood (~0 CFU at 2 h; Fig. 2E). The hemolytic activity of each locked SpnIII strain and the WT strain was also assessed, using purified human erythrocytes (O+) as the model. The strain expressing SpnIII allele B caused the highest amount of hemolysis (Fig. 2F and G). As hemolysis has been associated with both auto- and passive-lysis of pneumococci, we examined expression of the major autolysis associated gene—*lytA*. *lytA* expression was compared in each of the WT and locked strains during log-phase growth. A lower than 2-fold difference of *lytA* expression was found between all locked strains (Fig. 2H), suggesting the differences in hemolysis are due to passive lysis, not *lytA*-mediated autolysis. All strains also expressed pneumolysin, the major toxin produced by pneumococci at similar levels, indicating that rates of hemolysis were affected by unknown factors regulated by the SpnIII phasevarion.

### The SpnIII phasevarion impacts expression of conserved protein antigens.

**FIG 3:**
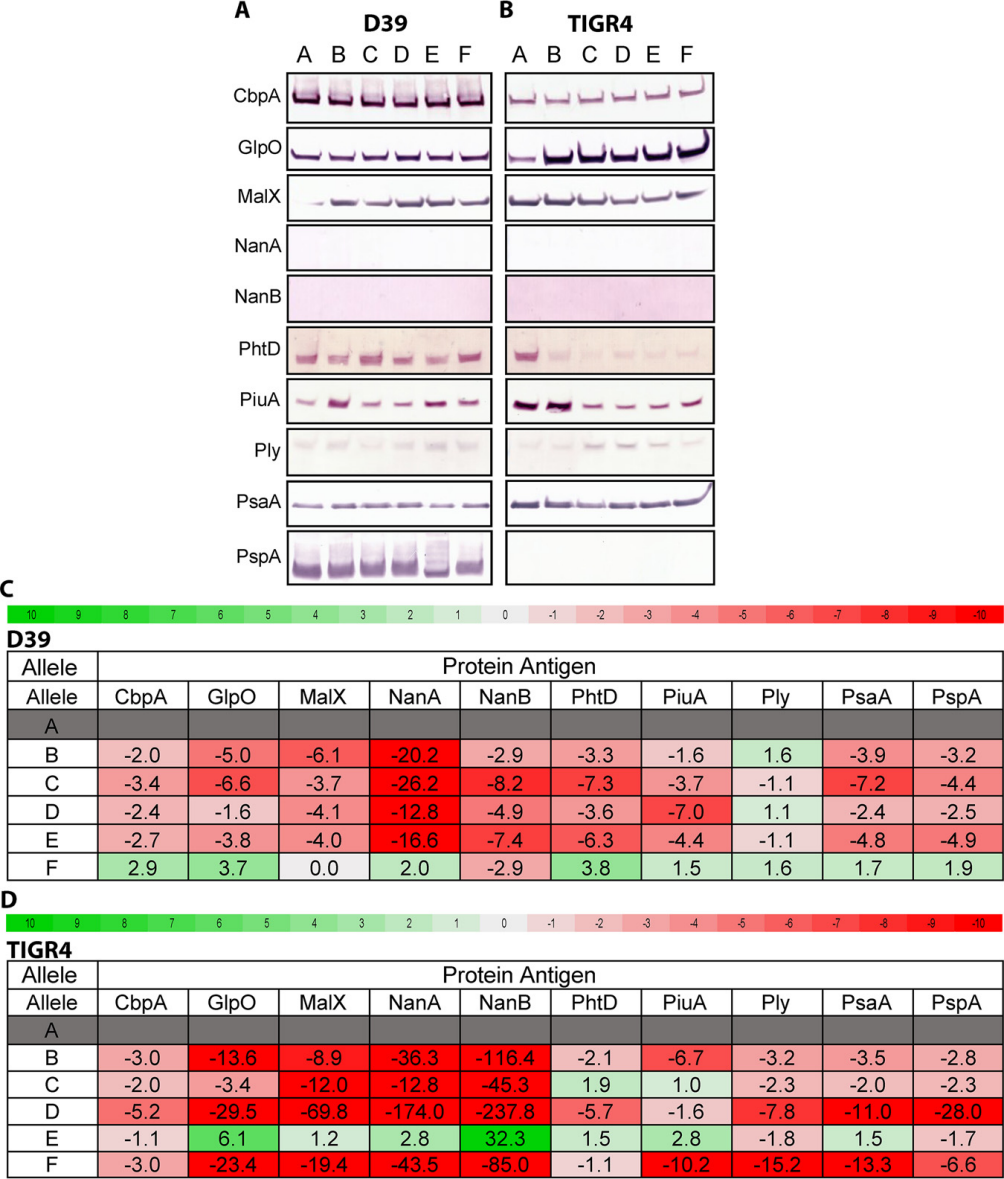
Expression differences in conserved proteinaceous vaccine candidates. Western blots examining differences in expression of protein levels between strains expressing alleles of the SpnIII system in strains (A) D39 and (B) TIGR4. Heatmaps of RT-qPCR expression data of concomitant expressed RNA levels of genes encoding these vaccine candidates from strains (C) D39 and (D) TIGR4. Red (negative fold difference) to green (positive fold difference) using SpnIII locked allele A as the baseline. Differences over >2 fold are in bold. All data with all locked variants as the baseline is presented in the same format in Table S2.

To study the extent of SpnIII-mediated gene expression changes on conserved protein antigens, we studied expression of 10 conserved pneumococcal surface proteins in prototype pneumococcal strains D39 (serotype 2) and TIGR4 (serotype 4). We used both WT and the six locked SpnIII variants in both strains D39 (22) and TIGR4 (23). We chose 10 conserved proteins that have been investigated as vaccine candidates: CpbA, GlpO, MalX, NanB, NanA, PhtD, PiuA, Ply, PsaA, and PspA. Standardized lysate loads were assessed by Coomassie staining (Fig. S2B). Differences in protein level were observed for multiple surface proteins in both strains, (Fig. 3A and B), with little correlation between specific SpnIII alleles and the strain being examined, indicating that there are both SpnIII phasevarion and strain-specific influences for expression of many of these conserved proteins. To investigate further, we carried out RT-qPCR using mRNA prepared from the same cultures as those used to prepare cell lysates for Western blots (Fig. 3C, D; full data in Table S2A, 2B), Significant (>2) fold differences were found in expression of vaccine candidates between the locked and WT strains, in both D39 and TIGR4. Gene expression of all vaccine candidates varied between locked strains. There was little correlation between Western blot differences and RT-qPCR expression data. To probe these differences further, whole-cell ELISAs were performed to examine the expression of targets that showed differential expression between Western blot and RT-qPCR (PiuA and PsaA) (Fig. S2C). We further quantified Western blot results (Fig. S2C) to evaluate fold difference of banding intensity, and for comparison to ELISAs. ELISAs showed protein expression profiles that aligned with the Western Blot, rather than the gene expression data. The inconsistency between mRNA and protein levels is not unusual, and has been seen in other studies (27). This indicates that SpnIII-mediated gene expression changes, coupled with strain/serotype specific differences, play a complex role in the regulation of expression of these conserved protein antigens.

### SpnIII-mediated capsule level differences are more important for pneumococcal survival than protein antigen levels.

**FIG 4:**
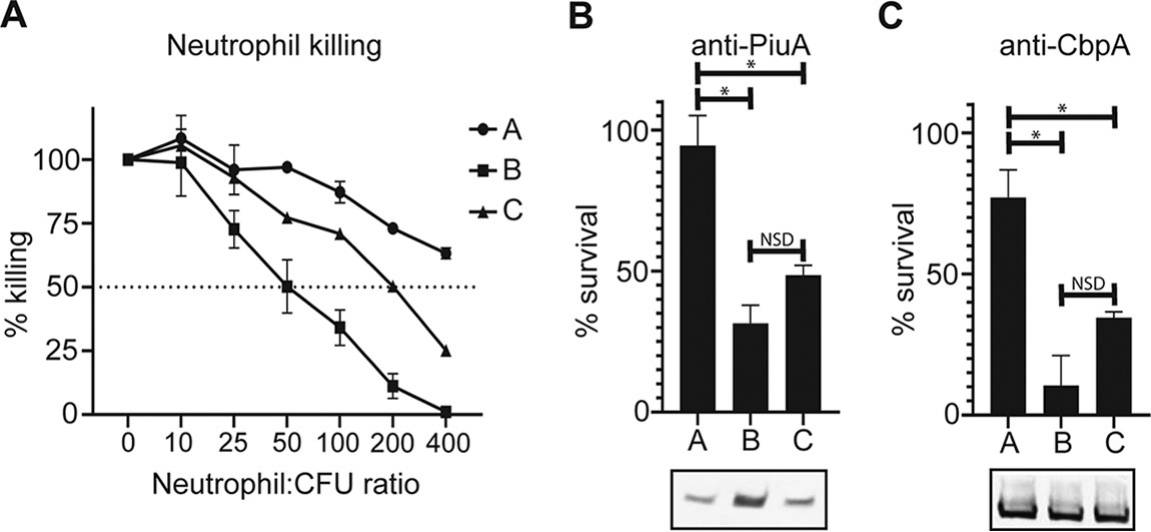
(A) Killing by differentiated neutrophil-like HL-60 cells with 10% (vol/vol) complement after 1 h. SpnIII A failed to reach 50% killing at maximum neutrophil:CFU ratio (400:1) after 1 h. SpnIII B and SpnIII C reached 50% killing at 50 and 200 neutrophil:CFU ratios, respectively. Opsonophagocytic killing assays using antisera against (B) PiuA and (C) CbpA at 1 in 20 dilution. Effects of antibody-mediated killing were masked by large differences in antibody-independent killing (A), independent of differences in antisera target. Western Blot showing expression of antisera targets in locked SpnIII alleles included for comparison. MOI for assays presented in Fig. S1. *, *P < *0.05; NSD, no significant difference.

Phenotypic differences between pneumococcal strains impact complement deposition and opsonophagocytosis (28, 29). We have shown the SpnIII variants (A to F) have different levels of protein expression of several putative protein vaccine candidates (Fig. 3). The SpnIII system has previously been shown to influence capsule level (22), with strains expressing SpnIII allele B showing the lowest level of capsule expression due to a downregulation of the *cps* locus in our SpnIII B locked variant (22). Capsular serotype/level have also been shown to impact complement resistance and opsonophagocytosis (30). We investigated if SpnIII phasevarion-mediated differences in both capsule quantity and protein antigen levels impacted *in vitro* opsonophagocytosis and killing by differentiated (neutrophil-like) HL-60 cells. We selected three of our D39 locked strains (locked for SpnIII A, B, and C) for their differences in capsule quantity; a D39 strain expressing SpnIII allele A has a high level of capsule relative to SpnIII allele B, with a strain expressing SpnIII allele C having an intermediate level of capsule compared with A and B (22). SpnIII allele A (high capsule phenotype) is much more resistant to opsonophagocytic killing compared with SpnIII allele B (low capsule phenotype) and SpnIII C (medium capsule phenotype) (Fig. 4A). We could not achieve 50% killing of the strain locked for SpnIII allele A, even at our maximum threshold of a 400:1 MOI (neutrophil:CFU). Study achieved 50% killing of SpnIII allele C with an MOI of 200:1 and SpnIII allele B with an MOI of 50:1. SpnIII allele B is over four times more susceptible to serum-independent neutrophil killing than SpnIII allele A. To investigate the effect of protein expression differences we observed in Fig. 3, we used PiuA and CbpA antisera in opsonophagocytosis assays (OPKs) (Fig. 4B and C). Western blots showed variable expression of PiuA commensurate with different SpnIII allele expression (*different* protein antigen level, different capsule level), whereas CbpA was equally expressed irrespective of the SpnIII allele expressed (*same* protein antigen level, different capsule level), allowing us to determine if capsule or protein antigen level was more important to survival. The large differences in antisera independent killing made comparison between locked alleles difficult. However, changes in protein antigen level impacted opsonophagocytosis less significantly than capsule level, with survival proportional to capsule amount (A > C>B) irrespective of protein antigen level.

## DISCUSSION

Phasevarions offer a contingency strategy for survival in many bacteria (15, 16, 31). In this study, we have built on our and others characterization of the SpnIII phasevarion (22–24). The “full” six-way SpnIII phasevarion was the most prevalent form, found in 56/82 of fully annotated S. pneumoniae genomes examined, indicating a key role of this system in pneumococcal pathobiology. However, we did identify variations on this system. The majority of the strains not producing a full six-way SpnIII phasevarion (24 strains) all encode systems that switch between two, three, or four SpnIII alleles (various combinations of existing alleles A to F). Five strains, such as strain 4041STDY6836167, appeared to have an SpnIII system which contained additional copies of the methyltransferase, and a total of nine *hsdS* regions (Table S1), including TRDs that have not been identified before. Duplicate SpnIII systems were also observed in ~6% of genomes (five strains). As only the six-way switch has been previously described in literature, and in only three strains (D39, TIGR4, and ST556), the impact of the truncated SpnIII phasevarions (not encoding all six HsdS alleles), the significance of duplications of the SpnIII system, and the role, if any, of newly identified TRDs, remains unclear.

We have shown that the full SpnIII phasevarion of S. pneumoniae produces phenotypically distinct sub-populations which have varied pathogenic phenotypes. It is therefore likely that synergy between the different phenotypes produced by the SpnIII phasevarion exists in a pneumococcal population, with each variant providing unique advantages (and disadvantages) under different conditions. These variants are likely subject to selection and counter-selection in different host niches dependent on the phenotypes they confer. There may be beneficial interactions between individual bacterial cells expressing different alleles in the pneumococcal population, with significantly more work needed to dissect these. However, our work has begun to determine the impact of each SpnIII allele on pneumococcal phenotype on a broad population level. For example, in strain D39, SpnIII allele B produces a phenotype that is more adherent to, and invasive of, host cells, perhaps providing a mechanism for intracellular survival and persistence (32). This property, however, comes at the expense of survival in blood, and an increased susceptibility to neutrophil-mediated killing (Fig. 4A), with both the positive and negative phenotypes the result of much decreased capsule expression (22). Lower capsule expression in cells expressing allele B also likely affects factors like shedding (33) and clearance (34). We see an opposite phenotype influenced by SpnIII allele A which expresses a high amount of capsule; strains expressing this allele show a much-decreased ability to form a biofilm, and reduced adherence to host cells (Fig. 2B to D), but shows high survival in blood (Fig. 2E), and is highly resistant to neutrophil killing (Fig. 4A). Individually, these phenotypes provide several advantages and disadvantages, but when produced in synergy they would provide a pneumococcal population with contingencies to survive in multiple host niches, and to rapidly respond to varying conditions. Diverse effects on pneumococcal phenotype were also seen previously. For example, biofilm formation was influenced with our locked TIGR4 strains (23); in strain ST556, host cell adherence and nasopharyngeal colonization were influenced by different SpnIII alleles (24).

We have shown that there are significant differences in hemolytic ability between the phenotypes produced by the SpnIII phasevarion during log-phase growth, independent of *lytA* expression. Haemolysis has been associated with both auto- (LytA-mediated), and passive-lysis (35–38) in S. pneumoniae. LytA is the major S. pneumoniae autolysin (35), and promotion of autolysis has been proposed to mediate release of virulence determinants such as Ply (39), and mediate S. pneumoniae fratricide (38). We did not see a difference in *lytA* expression between SpnIII alleles, indicating LytA production is not the cause of hemolytic differences between strains expressing different SpnIII alleles. Therefore, the differences seen in hemolytic activity may be primarily from passive pneumococcal lysis, and the exact factors responsible for this need to be determined. We also did not see a difference in pneumolysin expression (Fig. 3), discounting release or level of this toxin from the differences in hemolytic ability. Manso et al. reported the pneumococcal capsule was significantly lower in cells expressing SpnIII alleles B and C (B < C) (22) in strain D39, and we have shown this correlates with phenotypes that can be attributed to decreased capsule. Strains expressing alleles B and C demonstrate the highest levels of hemolysis. As capsule provides structural integrity to the cell, perhaps it is reduced capsule, controlled by SpnIII phase variation, that produces variants that are more prone to passive lysis.

We observed differences in vaccine candidate expression between our “locked” SpnIII strains in two diverse pneumococcal strains. Protein levels were evaluated via Western Blot (Fig. 3A to C) and results were correlated with whole cell ELISA (Fig. S2C). There were some vaccine candidates that had relatively equal expression between alleles, such as PsaA in D39, with the largest difference of 1.5 fold between allele D and allele E (Fig. S2C). However, strain D39 showed different expression of MalX, PhtD, and PiuA between SpnIII alleles. Quantification of the Western Blot revealed that, PiuA, for example, had a 2.7-fold difference between D39 allele B versus allele A (Fig. S2C). GlpO, PhtD, Ply, and PiuA protein levels showed clear expression differences in strain TIGR4. Differences seen between D39 and TIGR4 are likely due to a combination of effects, such as individual expression of different regulators/genes between the two strains, and from the proteins themselves differing between strains (e.g., D39 and TIGR4 had different families of PspA) (40, 41). Additional factors observed to vary due to the effects of the SpnIII phasevarion, such as capsule expression (22, 23), will also affect level of cell surface proteins (34, 42). The variation of protein antigen levels observed due to the SpnIII system may provide S. pneumoniae multiple contingency phenotypes, which are dynamically selected for/against depending on factors like co-colonization and immune detection, as has been seen with phasevarions in other host-adapted pathogens (18–20, 43). Previous RNA-Seq analysis using these locked strains in D39 (22) and TIGR4 (23) showed that only PsaA was previously identified in D39 by RNA-Seq as being differentially regulated. There is also low consistency between our RT-qPCR results and our Western blots, although in general where a gene is shown as upregulated by RT-qPCR, we see the same upregulation in our Western blotting, and so on. In general, it is accepted that there is low correlation between transcriptomic and proteomic analysis of gene expression in numerous other bacterial species (44–47). As such, significant further work is required to understand the precise ways these genes are regulated in diverse strains of S. pneumoniae, and we demonstrate that the SpnIII system should be taken into account when doing so.

Although differences in pneumococcal genotype have been seen to impact complement deposition and opsonophagocytosis (48), capsular serotype has been observed to be the primary determinant of complement-mediated killing (30, 49). Complicating this, the SpnIII system generates phenotypically distinct intra-strain sub-populations, which have been shown to have varied levels of pneumococcal capsule (22, 23). We have demonstrated that even within a single strain (D39; serotype 2), the differences in capsule due to SpnIII allele switching impact neutrophil killing and likely cellular adherence and invasion (Fig. 3 and 4). Our results are in agreement with the findings of Zangari et al., who recently found immunization against conserved surface proteins induced antibody titers unable to prevent colonization by an encapsulated strain, but were able to protect against an acapsular mutant of that same strain (that had comparable levels of antibody targets) (50). Our findings suggest there is a particular, specialized role for a variant expressing decreased capsule level (SpnIII B). It is possible that the protective effect seen by Zangari et al. (50) when targeting conserved surface proteins in an acapsular mutant would also be effective against naturally occurring SpnIII alleles with reduced capsule (such as SpnIII B and C in strain D39). Furthermore, targeting naturally occurring phenotypes that we have shown to be important in biofilm formation and cellular adherence and invasion may prove effective in preventing key stages of S. pneumoniae disease progression, such as colonization. Several of the vaccine targets we examined (PspA, PhtD, and Ply) are also known to inhibit C3 deposition (51) and interfere with complement-mediated killing. These proteins are differently expressed due to switching of the SpnIII phasevarion (Fig. 3), demonstrating that further work is required to fully characterize the effects of the SpnIII phasevarion on killing by the host immune system.

In conclusion, we have demonstrated that clinically relevant phenotypes (Fig. 2) conserved protein antigens (Fig. 3), and opsonophagocytic killing (Fig. 4) are all influenced by the SpnIII phasevarion, with many attributable to the effects of the SpnIII phasevarion on the level of capsule, correlating with previous findings (22, 23). Significantly, we have shown that the SpnIII phasevarion influences expression of conserved protein antigens, both within individual strains, and between diverse pneumococcal strains. This is of concern to development of a universal pneumococcal vaccine based on these antigens; although these proteins are highly conserved between pneumococcal strains, their regulation appears to be profoundly complex. Understanding how expression of these antigens is regulated is critical to the rational design of a universal pneumococcal vaccine, as alteration of expression of vaccine candidates could lead to a decrease in vaccine efficacy, and possibly even vaccine failure.

## MATERIALS AND METHODS

### Bacterial strains and growth conditions.

S. pneumoniae strains were derived from strain D39 (serotype 2) (22) or TIGR4 (serotype 4) (23) as described previously. S. pneumoniae was grown in Todd Hewitt broth supplemented with 1% yeast (THB+Y) at 37°C. Columbia 5% blood agar (CBA) plates used to grow S. pneumoniae on solid media at 37°C 5% CO_2_ conditions.

### Adherence assay.

S. pneumoniae adherence and invasion was assessed against human derived A549 cells (type II pneumocytes). ~10^5^ A549 cells were seeded to each well of a flat-bottomed 24-well plate (Greiner, Germany) and allowed to settle overnight (37°C) before inoculating with S. pneumoniae SpnIII variants (A to F) and wild-type D39 at ~10^7^ CFU per well (MOI of 100:1) in 250 μL of RPMI media (Dubco) 10% fetal calf serum. MOI ratios of A549 adherence and invasion are supplied in Fig. S1A. Plates were incubated for 1 h at 37°C 5% CO_2_ allowing adherence/invasion. For the adherence assay, wells were washed of all non-adherent S. pneumoniae via multiple, gentle, 1 mL washes of phosphate-buffered saline (PBS). Visual checks were performed to ensure A549s were intact, and planktonic S. pneumoniae removed. Wells were then detached via 250 μL 0.25% Trypsin EDTA to dislodge adherent cells (5 min, 37°C) before serial dilution and drop plating on CBA plates to enumerate bacterial loads. Results represent triplicate values of biological duplicates.

### Adherence to differentiated human airway epithelial cells.

S. pneumoniae adherence was assessed using normal human nasal airway epithelial (HNAE) cells differentiated *ex vivo*. These primary cells were differentiated *ex vivo* into basal cells, ciliated cells, and mucous-producing cells organized in a pseudostratified epithelium that replicates the structure and nature of the human upper airway epithelium. HNAE cells were collected from healthy donors (human ethics approval GLY/01/15/HREC) and expanded using Pneumacult Ex+ (Stemcell technologies). HNAE cells were differentiated at air-liquid interface in 6.5 mm transwell with a 0.4 μm polyester 156 membrane (Corning, Product No. 3470). Briefly, media was removed from HNAE cells apical side (airlift) and provided with Pneumacult ALI basal media from HNAE cells basolateral side (Stemcell technologies). HAE cells were fully differentiated and ready to use after 28 days postairlift. Airway cells were washed twice with prewarmed PBS to remove mucous (20 min, 37°C) prior to use. The amount of total HNAE cells per transwell was enumerated, with one fourth of the total cells expected to be on the apical surface—this one fourth value was used to calculate the MOIs. Mid-log cultures of locked SpnIII alleles A, B, and C from strain D39 were used to inoculate the airway cells at a surface MOI of ~5:1 in a minimal 50 μL volume of media alone (Dubco). MOI ratios provided in Fig. S1B CFU inputs were assessed at both initial inoculum and after 1 h at 37°C to monitor changes in CFU during experimentation. Plates were incubated for 1 h at 37°C with 5% (vol/vol) CO_2_. Wells were washed of non-adherent cells via multiple, gentle, washes with 200 μL of pre-warmed PBS. Wells were then treated with 200 μL of 0.25% Trypsin-EDTA to dislodge adherent bacteria (30 min 37°C) before serial dilution and drop plating on CBA plates to enumerate bacteria. The percentage adherence was calculated from the CFU in the inoculum.

### Invasion assay.

The invasion assay was identical to adherence assay (A549s) with the following changes: A549s were incubated with S. pneumoniae variants for 1 h, before extracellular bacteria were killed via treatment with 50 μg/mL Pen G in RPMI 10% foetal calf serum (FCS) for 45 min at 37°C. Wells were then treated with 250 μL of 0.25% trypsin EDTA to detach A549s (5 min 37°C). Wells were then treated with 0.4% Saponin to lyse A549s (releasing invasive bacteria). Visual checks made to confirm cell lysis. Controls were in place to ensure effectiveness of Penicillin G treatment (data not shown). Surviving intracellular pneumococci were enumerated via serial dilution and drop plating on CBA plates. Results represent triplicate values of biological duplicates.

### Biofilm formation.

Log-phase S. pneumoniae locked alleles/wild-type D39 was resuspended to OD 0.05 in THB+Y. Flat-bottomed 24-well plates (Greiner, Germany) were seeded with 1 mL inoculum and grown for 24 h (37°C 5% CO_2_). Wells stained and fixed with 0.5% crystal violet for 15 min. Planktonic bacteria were removed via PBS washes. Remaining biomass measured by resuspending crystal violet with 95% ethanol and taking absorbance at 590 nm. Results represent triplicate values of biological duplicates.

### Survival in whole blood.

Fresh human whole blood was drawn from volunteers into heparin-coated tubes by a registered nurse at Griffith University. Blood was held at 37°C, with shaking, and used within 2 h of the initial bleed. Experiments were carried out in triplicate on two separate occasions. Using a 96-well flat-bottomed plate (Greiner, Germany), 200 μL of whole human blood was inoculated with ~5 × 10^5^ CFU of strains expressing alleles A to F and wild-type S. pneumoniae (Fig. S1D). Inoculum CFU were quantified by serial dilution to ensure equal loading of each well. After 90 min incubation at 37°C, shaking (120 rpm), with output CFU enumerated by serial dilution and drop plating. The percent survival was calculated by dividing the surviving cells in blood (output) versus inoculation dose (input). Data represents triplicate values from biological duplicates.

### Haemolysis assay.

Using 96-well round-bottomed plates (Greiner, Germany), 200 μL of 10% purified human erythrocytes (Red Cross Blood Service, Australia) suspended in PBS was inoculated with 0.22u filtered minimal media of S. pneumoniae “locked” strains expression SpnIII alleles A to F and wild-type D39. Chemically defined media (CDM) was prepared using SILAC RPMI (no glucose, no phenol red; Life Technologies Australia) 5% glucose and prepared as per Minhas et al. (52). Erythrocytes were resuspended in dilutions ranging from neat media down to a 1/100 dilution of media and PBS. Plates were incubated at 37°C, shaking at 200 rpm (lid on) for 1 h. Plates were removed and centrifuged at 1,000 rpm for 10 min to pellet surviving erythrocytes. Then, 100 μL of supernatant was moved to a 96-well flat-bottomed plate (Greiner, Germany) and read at an absorbance of 540 nm to detect amount of free haem and, indirectly, amount of lysis. No difference in absorbance observed between filtered minimal media and blank PBS. A PBS solution of 5% Saponin was used to produce 100% erythrocyte lysis, which was used as a standard for the amount of lysis of the S. pneumoniae media. Results represent triplicate values.

### Protein extraction.

To compare surface bound proteins of SpnIII variants, protein was extracted from log-phase bacteria. Briefly, bacteria were grown to OD600 0.8 in TBH+Y, representing late-log phase growth. Then, 10 mL of this was pelleted and washed with THB+Y. Bacterial pellet was then resuspended in 1.5 mL RIPA Buffer (53) before sonicating for 30 s three times, resting for 2 min between bursts. Bacteria and debris were pelleted out and supernatant (containing protein) taken. Protein lysates were stored at −20°C with 10% β-mercaptoethanol.

### Western blotting.

Protein load was standardized by visual checks on a 12% Bis-Tris Gel (Fig. S2B). Gels were run at 150V for 45 mins in Bolt MOPS Running Buffer (Invitrogen). Protein in lysates was transferred to nitrocellulose membrane at 15V for 1 h. Nitrocellulose membranes were blocked with 5% (wt/vol) skim milk in Tris-buffered saline with 0.1% Tween 20 (TBST) by shaking overnight at 4°C. Primary mouse antibodies against S. pneumoniae vaccine antigens were sourced from the Paton lab (Adelaide). Primary antibodies used at 1:100 to 1,000 in 5% skim milk TBST for 1 h with shaking at room temperature. Nitrocellulose membrane was washed in TBST for 1 h before adding secondary antibody (goat anti-mouse alkaline phosphatase conjugate; catalogue number A3562) (Sigma) in 5% skim milk TBST at 1:2,500 dilution. Membranes were washed for 1 h in TBST, before developing for 5to 10 min, or overnight at room temperature with shaking. Developing solution comprised of 100 mM Tris-HCL (pH 9.5), 100 mM NaCl, 5 mM MgCl_2_ with bromo-chloro-indolyl phosphate (BCIP), and nitro blue tetrazolium (NBT) used as detectors. In the case of PspA, antisera proved to be effective at detecting PspA of D39, which is classified as a family 1 PspA (54), but was infective at detecting the TIGR4 family 2 PspA (40) (evident by the lack of banding). Neither NanA nor NanB could be effectively detected in lysates after multiple attempts of varying conditions.

### S. pneumoniae RNA extraction.

Trizol protocol was followed for RNA extraction of Streptococcus spp. as per manufacturer’s instructions. Briefly, 2 mL of log-phase S. pneumoniae at OD600 0.8 was pelleted and treated with lysozyme and mutanolysin for 30 min at 37°C before adding 1 mL Trizol reagent and storing at −80°C. Trizol RNA extraction carried out as per manufacturer’s instructions (Sigma).

### Quantitative real-time PCR.

Real-time PCRs were performed in triplicate using RNA isolated from “locked” SpnIII alleles A to F in strain D39 and TIGR4, as described above. cDNA was synthesized using NEB Protoscript II and random hexamers (Invitrogen; 50 ng μL^−1^) according to the manufacturer’s instructions. Reverse transcriptase reactions lacking Protoscript II were performed as a negative control. All real-time PCRs were performed in a 25-μL mixture containing a 1 in 5 dilution of the cDNA preparation (5 μL), 10xSYBR Green buffer (PE Applied Biosystems) and 2 μM each primer (see Table S3 for primers). Pneumococcal 16S RNA was used as a control in each quantitative PCR comparison. Amplification and detection of specific products were performed with the ABI Prism 7700 sequence-detection system (PE Applied Biosystems) with the following cycle profile: 95°C for 10 min, followed by 40 cycles of 95°C for 30 s and 60°C for 1 min. The data was analyzed with ABI prism 7700 (version 1.7) analysis software. Relative gene expression between samples was determined using the ^ΔΔ^CT relative quantification method.

### Opsonophagocytic killing assays.

HL60 cells were differentiated in DMSO (0.8%) containing M2 media (RPMI 1640, 10% FBS, 1% l-glutamine) for 6 days prior to use. To determine HL60 differentiation, flow cytometry analysis was carried out to determine >55% of cells expressing the maturation marker CD35 (E11, Bio-Rad) and >12% of cells expressing the proliferation marker CD71 (DF1513, Bio-Rad) (PMID: 32430834). Differentiated HL60s were activated in Hanks’ balanced salt solution (HBSS) containing Ca^++^ and Mg^++^ prior to use. The ability of the differentiated HL60 neutrophil-like cells to kill S. pneumoniae D39 locked alleles A, B, and C was assessed across varying concentrations of rabbit complement and neutrophils with a range of 20% complement + 4 × 10^5^ Neutrophils per 1 × 10^3^ CFU of S. pneumoniae down to complement and neutrophil free media. Complement + media-only controls did not kill S. pneumoniae (data not shown). Killing was assessed after 1 h at 37°C in conditions with and without mouse antisera. Results were expressed as percent of the surviving inoculum in the control wells for the neutrophil killing assay, and percent survival of the starting inoculum (~1,000 CFU) in the opsonophagocytosis assays. Input amounts of each Locked allele can be seen in Fig. S1C. Graphs and statistics were generated via GraphPad Prism 5.0 (GraphPad Software, La Jolla, CA). Error bars represent standard deviation from mean values. Student’s *t* test was used to compare samples: *P* values of <0.05 (considered significant) represented by *, *P* value of <0.001 indicated by **, *P* value of <0.001 indicated by ***. Groups were considered not significantly different if *P* > 0.05 (no *).

### Data availability.

This manuscript has been deposited in BioRxiv: https://www.biorxiv.org/content/10.1101/2022.02.08.479631v1.
