# Supplementary material for: Pneumococcal Phasevarions Control Multiple Virulence Traits, Including Vaccine Candidate Expression
Source: Microbiol Spectr. 2022 May 10;10(3):e00916-22. doi: 10.1128/spectrum.00916-22 (PMC9241608; doi:10.1128/spectrum.00916-22)
Supplement: SUPPLEMENTAL FILE 1 — Supplemental material. Download spectrum.00916-22-s001.pdf, PDF file, 1.4 MB [file spectrum.00916-22-s001.pdf]

Supplementary Tables

A

Strain	SpnIII System Genetic Region									Methyltransferase	Type	Serotype	Sequence Type
	1	2	3	4	5	6	7	8	9				
310	1.1>	1.2>	**PsrA>**	2.3>	<2.2	<1.1				<M	3	24F	11618
335	1.1>	2.3>	<2.2	**<PsrA**	<2.1	<1.2				<M	1	19F	81
475	1.1>	1.2>	<2.2	**<PsrA**	<2.1	<1.2				<M	3	24F	15253
521	1.2>	2.1>	**PsrA>**	2.3>	<2.2	<1.1				<M	1	24F	15253
521_2	1.2>	2.1>	**PsrA>**	2.2>	<2.3	<1.1				<M	1	24F	15253
525	1.1>	2.1>	**PsrA>**	2.3>	<2.2	<1.1				<M	1	24F	15253
563	1.2>	2.1>	**PsrA>**	2.2>	<2.3	<1.1				<M	1	24F	15253
566	1.1>	2.1>	**PsrA>**	2.3>	?	<1.2				<M	1	24F	11618
573	1.2>	2.2>	<2.3	**<PsrA**	<2.1	<1.1				<M	1	24F	14184
574	1.2>	2.1<	**PsrA>**	2.3<	<2.2	<1.1				<M	1	24F	11618
947	1.1>	2.1>	**PsrA>**	?	?	<1.1				<M	3	4	15
4496	1.2>	2.1>	**PsrA>**	2.3>	<2.2	<1.1				<M	1	1C	3018
70585	1.1>	2.3>	<2.2	<1.2						<M	2	5	289
11A	1.2>	2.1>	**PsrA>**	2.3>	<2.2	<1.1				<M	1	11A	8279
180-15	1.2>	2.3>	<2.2	**<PsrA**	<2.1	<1.1				<M	1	3	180
180-2	1.1>	2.2>	<2.3	**<PsrA**	<2.1	<1.2				<M	1	3	180
19A-19339	1.2>	2.3>	<2.2	<1.2						<M	4	19A	unidentified
2245STDY6178787	1.2>	2.1>	**PsrA>**	2.3>	2.2>	<2.3	<1.1			<M	5	6B	315
4041STDY6583227	1.2>	2.1>	**PsrA>**	2.3>	<2.2	<1.1				<M	1	12F	unidentified
4041STDY6836166	<2.2	**<PsrA**	<2.1	<1.2						<M	5	16F	unidentified
4041STDY6836166_2	1.2>	2.1>	**<PsrA**	2.2>	2.3>	<1.2				<M	3	16F	unidentified
4041STDY6836167	2.2>	<2.3	M>	1.2>	2.2>	<2.3	**<PsrA**	<2.1	<1.2	<M	5	9V	unidentified
4041STDY6836169	1.2>	2.1>	**PsrA>**	2.3>	<2.2	<1.1				<M	1	6B	unidentified
4041STDY6836169_2	1.1>	2.1>	**PsrA>**	2.2>	<2.3	<1.1				<M	3	6B	unidentified
4041STDY6836170	1.2>	2.2>	<2.3	**<PsrA**	<2.1	<1.2				<M	3	7C	unidentified
670-6B	1.2>	2.3>	<2.2	**<PsrA**	<2.1	<1.1				<M	1	6B	90
6A-10	1.2>	2.1>	**PsrA>**	2.3>	<2.2	<1.1				<M	1	6A	460
A45												3	6934
A66												3	378
AP200	1.2>	2.2>	<2.3	**<PsrA**	<2.1	<1.1				<M	1	11A	62
ASP0581	1.1>	2.3>	<2.2	**<PsrA**	<2.1	<1.2				<M	1	12F	4846
ATCC 49619	1.2>	2.1>	**PsrA>**	2.3>	<2.2	<1.1				<M	1	19F	1203
ATCC 700669	1.1>	2.3>	<2.2	**<PsrA**	<2.1	<1.2				<M	1	23F	81
AUSMDU00010538	1.2>	2.1>	**PsrA>**	?	?	<1.1				<M	1	19A	199
B1900	1.2>	2.2>	<2.3	<1.1						<M	2	3	unidentified
BHN97x	1.2>	2.1>	**PsrA>**	2.3>	<2.2	<1.2				<M	3	19F	425
BVJ1JL	1.2>	2.2>	<2.3	**<PsrA**	<2.1	<1.2				<M	3	1	unidentified
CGSP14	1.1>	2.3>	<2.2	<1.2						<M	2	14	15
D39	1.2>	2.2<	<2.3	**<PsrA**	<2.1	<1.1				<M	1	2	595
EF3030	?	2.1>	**PsrA>**	?	?	<1.1				<M	1	19F	43
FDAARGOS-1508	1.2>	2.1>	**PsrA>**	2.2>	<2.3	<1.1				<M	1	unidentified	unidentified
G54	1.1>	2.2>	<2.3	<1.1						<M	4	19F	NA*
gamPNI0373	1.2>	2.1>	**PsrA>**	2.3>	<2.2	<1.2				<M	3	1	618
GPSC10	1.2>	2.1>	?	?						<M	2	24F	2013
GPSC10_2	1.2>	2.2>	<2.3	**<PsrA**	<2.1	<1.1				<M	1	24F	2013
GPSC18	2.2>	<2.3	?	**<PsrA**	<2.1	<1.1				<M	1	14	13
HKU1-14	1.2>	2.2>	<2.3	**<PsrA**	<2.1	<1.2				<M	3	unidentified	6011
Hungary19A-6	1.2>	2.1>	**PsrA>**	2.3>	<2.2	<1.1				<M	1	19A	268
HU-OH	?	<1.1								<M	5	3	180
INV104	1.2>	2.3>	<2.2	<1.1						<M	2	1	227
INV200	?	2.1>	**PsrA>**	2.3>	<2.2	<1.1				<M	1	14	9
JJA	1.2>	2.3>	<2.2	**<PsrA**	<2.1	<1.1				<M	1	19F	66
KK0981	1.2>	2.1>	**PsrA>**	2.3>	<1.2	<1.1				<M	3	3	242
M16808	1.1>	2.1>	**PsrA>**	2.2>	<2.3	<1.2				<M	1	unidentified	unidentified
M23734	1.2>	2.1>	**PsrA>**	2.3>	<2.2	<1.2				<M	3	unidentified	unidentified
M26365	1.2>	2.2>	<2.3	**<PsrA**	<2.1	<1.1				<M	1	unidentified	unidentified
M26368	1.1>	2.1>	**PsrA>**	2.3>	<2.2	<1.2				<M	1	unidentified	unidentified
MDRSPN001	1.1>	2.2>	<2.3	**<PsrA**	<2.1	<1.2				<M	1	19F	10017
NCTC11902	1.2>	2.1>	**PsrA>**	2.3>	<2.2	<1.1				<M	1	14	124
NCTC12977	1.1>	2.1>	**PsrA>**	2.2>	<2.3	<1.2				<M	1	19F	1203
NCTC12977_2	1.1>	2.1>	**PsrA>**	2.2>	<2.3	<1.2				<M	1	19F	1203
NCTC13276	1.2>	2.2>	<2.3	**<PsrA**	<2.1	<1.1				<M	1	NE	595
NCTC7465	1.2>	>2.3	<2.2	**<PsrA**	<2.1	<1.1				<M	1	1	615
NCTC7465_2	1.2>	2.1>	**<PsrA**	2.3>	<2.2	<1.1				<M	1	1	615
NCTC7466	1.2>	2.2>	<2.3	**<PsrA**	<2.1	<1.1				<M	1	2	595
NT-110-58	1.1>	2.1>	**PsrA>**	2.2>	<2.3	<1.2				<M	1	NE	344
NU83127	1.1>	2.1>	**<PsrA**	2.2>	<2.3	<1.1				<M	3	4	246
OXC141	1.2>	2.3>	<2.2	**<PsrA**	<2.1	<1.1				<M	1	3	180
P1031	1.2>	2.1>	**PsrA>**	2.3>	<2.2	<1.2				<M	3	1	303
PZ900701590	1.1>	2.1>	**PsrA>**	2.3>	<2.2	<1.1				<M	3	12F	6945
R36A	1.1>	2.2>	<2.3	**<PsrA**	<2.1	<1.2				<M	1	NE	595
R6	1.2>	2.2>	<2.3	**<PsrA**	<2.1	<1.1				<M	1	NE	595
R6CIB17	1.2>	2.2>	<2.3	**<PsrA**	<2.1	<1.1				<M	1	NE	595
Rx1	1.2>	2.1>	**PsrA>**	2.3>	<2.2	<1.1				<M	1	NE	595
SCAID PHRX1-2021	1.2>	2.1>	**PsrA>**	2.3>	<2.2	<1.1				<M	1	unidentified	377
SNP034156	1.2>	2.1>	**PsrA>**	2.2>	<2.3	<1.1				<M	1	3	180
SP49	1.2>	2.1>	**PsrA>**	2.2>	<2.3	<1.1				<M	1	19A	277
SP61	1.2>	2.1>	**PsrA>**	2.2>	<2.3	<1.1				<M	1	19A	2432
SP64	1.2>	2.1>	**PsrA>**	2.2>	<2.3	<1.1				<M	1	19A	320
SPN XDR SMC1710-32	**<PsrA**	<2.1	<1.1							<M	5	15A	8279
SPN032672	1.2>	2.3>	<2.2	<1.1						<M	2	1	unidentified
ST556	1.2>	2.1>	**PsrA>**	2.2>	<2.3	<1.1				<M	1	19F	1392
SWU02	1.1>	2.1>	**PsrA>**	2.2>	<2.3	<1.2				<M	1	19F	unidentified
Taiwan19F-14	1.2>	2.2>	<2.3	<1.1						<M	2	19F	236
TCH8431/19A	1.2>	2.1>	**PsrA>**	2.3>	<2.2	<1.1				<M	1	19A	320
TIGR4; ATCC BAA-334	1.2>	2.1>	**PsrA>**	2.3>	<2.2	<1.1				<M	1	4	205
Xen35	1.2>	2.1>	**PsrA>**	2.3>	<2.2	<1.1				<M	1	4	205

B

SpnIII Type	Prevalence in Genomes
1	68.3%
2	8.5%
3	18.3%
4	2.4%
5	6.1%
Two SpnIII Systems	6.1%
Absent	2.4%

Supplementary Table 1. SpnIII systems in fully annotated NCBI *S. pneumoniae* genomes. **A)** Orientation and genetic features of SpnIII systems. Direction of each feature indicated with either > or <. *hsdS* sequences 1.1, 1.2, 2.1, 2.2 and 2.3 included. Red highlighted sections have low (<80%) sequence identity to known *hsdS* sequences. "?" indicates a *hsdS* region with an identity <50% to the known *hsdS* sequences. Duplications of features are indicated in Green. The CreX recombinase – Pneumococcal site-specific recombinase A (PsrA) included in **Bold**. The sequence for the associated Methyltransferase (M) was also included with orientation. Serotype and Sequence Type information for each strain have been included where available. *NA indicates the strain contains 6 of 7 MLST genes, and NE indicates the strain is non-encapsulated. The prevalence of each major type of SpnIII system found in genomes can be seen in **B)**. Type 1 is the 'traditional' six-way switch as seen in **Figure 1A**. Type 2, seen in **Figure 1B**, is a four-way switch. Type 3 is a three-way switch as per **Figure 1C**. Type 4 is a two-way switch, as per **Figure 1D**. Type 5 were instances that did not fall into the previous four categories.

A	Protein Antigen Expression in D39									
**Allele**	**CbpA**	**GlpO**	**MalX**	**NanA**	**NanB**	**PhtD**	**PiuA**	**Ply**	**PsaA**	**PspA**
A										
B	-2.0	-5.0	-6.1	-20.2	-2.9	-3.3	-1.6	1.6	-3.9	-3.2
C	-3.4	-6.6	-3.7	-26.2	-8.2	-7.3	-3.7	-1.1	-7.2	-4.4
D	-2.4	-1.6	-4.1	-12.8	-4.9	-3.6	-7.0	1.1	-2.4	-2.5
E	-2.7	-3.8	-4.0	-16.6	-7.4	-6.3	-4.4	-1.0	-4.7	-4.9
F	2.0	2.4	-1.1	1.4	-3.5	1.2	1.1	1.5	1.3	1.3

Allele	CbpA	GlpO	MalX	NanA	NanB	PhtD	PiuA	Ply	PsaA	PspA
A	2.0	5.0	6.1	20.2	2.9	3.3	1.6	-1.6	3.9	3.2
B										
C	-1.7	-1.3	1.6	-1.3	-2.8	-2.2	-2.4	-1.8	-1.8	-1.4
D	-1.2	3.0	1.5	1.6	-1.7	-1.1	-4.5	-1.5	1.7	1.3
E	-1.4	1.3	1.5	1.2	-2.5	-1.9	-2.8	-1.7	-1.2	-1.5
F	4.0	11.8	5.6	27.8	-1.2	4.0	1.8	-1.1	5.0	4.3

Allele	CbpA	GlpO	MalX	NanA	NanB	PhtD	PiuA	Ply	PsaA	PspA
A	3.4	6.6	3.7	26.2	8.2	7.3	3.7	1.1	7.2	4.4
B	1.7	1.3	-1.6	1.3	2.8	2.2	2.4	1.8	1.8	1.4
C										
D	1.4	4.0	-1.1	2.1	1.7	2.0	-1.9	1.2	3.0	1.8
E	1.3	1.8	-1.1	1.6	1.1	1.2	-1.2	1.1	1.5	-1.1
F	6.8	15.8	3.4	36.0	2.4	8.7	4.2	1.7	9.1	5.9

Allele	CbpA	GlpO	MalX	NanA	NanB	PhtD	PiuA	Ply	PsaA	PspA
A	2.4	1.6	4.1	12.8	4.9	3.6	7.0	-1.1	2.4	2.5
B	1.2	-3.0	-1.5	-1.6	1.7	1.1	4.5	1.5	-1.7	-1.3
C	-1.4	-4.0	1.1	-2.1	-1.7	-2.0	1.9	-1.2	-3.0	-1.8
D										
E	-1.1	-2.3	1.0	-1.3	-1.5	-1.7	1.6	-1.1	-2.0	-2.0
F	4.8	3.9	3.8	17.6	1.4	4.3	8.1	1.4	3.0	3.3

Allele	CbpA	GlpO	MalX	NanA	NanB	PhtD	PiuA	Ply	PsaA	PspA
A	2.7	3.8	4.0	16.6	7.4	6.3	4.4	1.0	4.7	4.9
B	1.4	-1.3	-1.5	-1.2	2.5	1.9	2.8	1.7	1.2	1.5
C	-1.3	-1.8	1.1	-1.6	-1.1	-1.2	1.2	-1.1	-1.5	1.1
D	1.1	2.3	1.0	1.3	1.5	1.7	-1.6	1.1	2.0	2.0
E										
F	5.3	9.0	3.7	22.8	2.1	7.5	5.0	1.6	6.0	6.6

Allele	CbpA	GlpO	MalX	NanA	NanB	PhtD	PiuA	Ply	PsaA	PspA
A	-2.0	-2.4	1.1	-1.4	3.5	-1.2	-1.1	-1.5	-1.3	-1.3
B	-4.0	-11.8	-5.6	-27.8	1.2	-4.0	-1.8	1.1	-5.0	-4.3
C	-6.8	-15.8	-3.4	-36.0	-2.4	-8.7	-4.2	-1.7	-9.1	-5.9
D	-4.8	-3.9	-3.8	-17.6	-1.4	-4.3	-8.1	-1.4	-3.0	-3.3
E	-5.3	-9.0	-3.7	-22.8	-2.1	-7.5	-5.0	-1.6	-6.0	-6.6
F										

B	Protein Antigen Expression in TIGR4									
**Allele**	**CbpA**	**GlpO**	**MalX**	**NanA**	**NanB**	**PhtD**	**PiuA**	**Ply**	**PsaA**	**PspA**
A										
B	3.0	13.6	8.9	36.3	116.4	2.0	6.7	3.2	3.5	2.7
C	2.0	3.4	12.0	12.7	45.3	-1.9	-1.0	2.3	2.0	2.3
D	5.2	29.5	69.8	174.0	246.8	5.7	1.6	7.8	11.0	27.9
E	1.1	-6.1	-1.2	-2.8	-32.3	-1.5	-2.8	1.8	-1.5	1.7
F	3.0	23.4	19.4	43.5	84.9	1.1	10.2	15.2	13.3	6.6
**Allele**	**CbpA**	**GlpO**	**MalX**	**NanA**	**NanB**	**PhtD**	**PiuA**	**Ply**	**PsaA**	**PspA**
A	-3.0	-13.6	-8.9	-36.3	-116.4	-1.7	-6.7	-3.2	-3.5	-2.7
B										
C	-1.5	-4.0	1.3	-2.8	-2.6	-3.2	-6.7	-1.4	-1.7	-1.2
D	1.7	2.2	7.8	4.8	2.1	3.3	-4.3	2.4	3.1	10.2
E	-2.7	-82.8	-10.3	-102.5	-3762.8	-2.6	-18.9	-1.8	-5.1	-1.6
F	1.0	1.7	2.2	1.2	-1.4	-1.5	1.5	4.7	3.8	2.4
**Allele**	**CbpA**	**GlpO**	**MalX**	**NanA**	**NanB**	**PhtD**	**PiuA**	**Ply**	**PsaA**	**PspA**
A	-2.0	-3.4	-12.0	-12.7	-45.3	3.5	1.0	-2.3	-2.0	-2.3
B	1.5	4.0	-1.3	2.8	2.6	7.1	6.7	1.4	1.7	1.2
C										
D	2.6	8.7	5.8	13.6	5.5	19.7	1.6	3.4	5.4	12.3
E	-1.8	-20.8	-13.8	-36.0	-1463.5	2.3	-2.8	-1.3	-2.9	-1.3
F	1.5	6.9	1.6	3.4	1.9	3.9	10.2	6.7	6.5	2.9
**Allele**	**CbpA**	**GlpO**	**MalX**	**NanA**	**NanB**	**PhtD**	**PiuA**	**Ply**	**PsaA**	**PspA**
A	-5.2	-29.5	-69.8	-174.0	-246.8	-5.7	-1.6	-7.8	-11.0	-27.9
B	-1.7	-2.2	-7.8	-4.8	-2.1	-2.8	4.3	-2.4	-3.1	-10.2
C	-2.6	-8.7	-5.8	-13.6	-5.5	-10.6	-1.6	-3.4	-5.4	-12.3
D										
E	-4.7	-179.7	-80.6	-491.0	-7981.1	-8.5	-4.4	-4.4	-16.0	-16.0
F	-1.7	-1.3	-3.6	-4.0	-2.9	-5.0	6.5	2.0	1.2	-4.2
**Allele**	**CbpA**	**GlpO**	**MalX**	**NanA**	**NanB**	**PhtD**	**PiuA**	**Ply**	**PsaA**	**PspA**
A	-1.1	6.1	1.2	2.8	32.3	1.5	2.8	-1.8	1.5	-1.7
B	2.7	82.8	10.3	102.5	3762.8	3.1	18.9	1.8	5.1	1.6
C	1.8	20.8	13.8	36.0	1463.5	-1.3	2.8	1.3	2.9	1.3
D	4.7	179.7	80.6	491.0	7981.1	8.5	4.4	4.4	16.0	16.0
E										
F	2.7	143.0	22.4	122.9	2747.0	1.7	28.7	8.6	19.3	3.8
**Allele**	**CbpA**	**GlpO**	**MalX**	**NanA**	**NanB**	**PhtD**	**PiuA**	**Ply**	**PsaA**	**PspA**
A	-3.0	-23.4	-19.4	-43.5	-84.9	-1.1	-10.2	-15.2	-13.3	-6.6
B	-1.0	-1.7	-2.2	-1.2	1.4	1.8	-1.5	-4.7	-3.8	-2.4
C	-1.5	-6.9	-1.6	-3.4	-1.9	-2.1	-10.2	-6.7	-6.5	-2.9
D	1.7	1.3	3.6	4.0	2.9	5.0	-6.5	-2.0	-1.2	4.2
E	-2.7	-143.0	-22.4	-122.9	-2747.0	-1.7	-28.7	-8.6	-19.3	-3.8
F										

Supplementary Table 2. Putative vaccine target RTqPCR. Heatmap of putative vaccine target gene expression across strains **A)** D39 and **B)** TIGR4 expressing individual SpnDIII alleles ranging from red

(- fold difference) to green (+ fold difference). Gene expression values from locked alleles in grey have been used as a baseline against other alleles.

Primer Name	Forward/Reverse	Sequence (5' - 3')
CbpA_RT_F	F	CAAGGTAAACCAAAGGGGCG
CbpA_RT_R	R	TCAGGGATGAGCTTGGAAGAG
GlpO_RT_F	F	CATTGCCAACCACGTGAAGG
GlpO_RT_R	R	AACCAGACGGGCCTTGATTT
MalX_RT_F	F	TACGCATTCGCTGGTGAAGA
MalX_RT_R	R	AGCGTCTTTACCGTTTTGGC
NanA_RT_F	F	ATGACGACGGGAAGACATGG
NanA_RT_R	R	TTGTGAGGCCCATTCCGAAG
NanB_RT_F	F	TCTAGAAGTGGCCGTAAGGGA
NanB_RT_R	R	AGGCATAACCATACGAAGGCAA
PhtD_RT_F	F	AGCTGTTCGAAAAGTAGGCGA
PhtD_RT_R	R	ACTTTCCTGCTTGGCCAGTT
Ply_RT_F	F	CCCACTCTTCTTGCGGTTGA
Ply_RT_R	R	TCCGCGAACACTTGAATTGC
PspA_RT_R	F	CGCTCCTCAAGCTAAAATCGC
PspA_RT_R	R	GAAGAGGAGCACGGAAACCT
PiuA_RT_F	F	CCGAAGGCACTTGCTAAGGA
PiuA_RT_R	R	TCGCTTCACCACGTACACAA
PsaA_RT_F	F	CAGCGACGGCGTTGATGTTA
PsaA_RT_R	R	TTGGCGCTCAATTGTTTGGC
lytA_RT_F	F	CGGTTGGAATGCTGAGACCT
lytA_RT_R	R	GGCAAACCTGCTTCATCTGC
T4_PspA_RT_F	F	CCAGCGTCGCTATCTTAGGG
T4_PspA_RT_R	R	TCTTGGCAGTATCAGCTTTTGC
T4_PhtD_RT_F	F	AGCAGTAGTTGCAGCCAGAG
T4_PhtD_RT_R	R	TAATGGTCGCCGTGAGGAAC
16s_RT_F	F	AACCAAGTAACTTTGAAAGAAGAC
16s_RT_R	R	AAATTTAGAATCGTGGAATTTTT

Supplementary Table 3. RTqPCR Primers.

Supplementary Figure 1

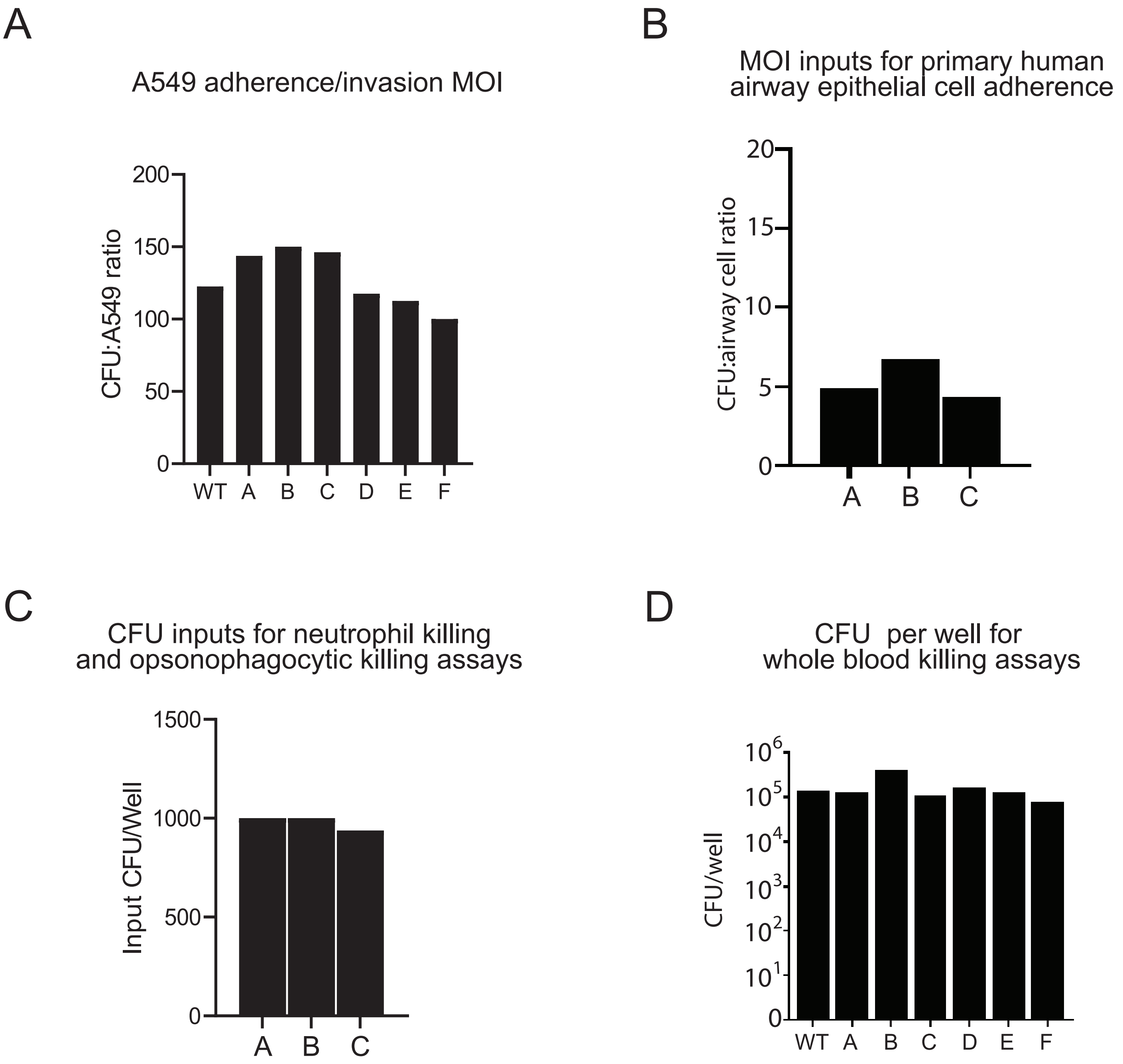

Supplementary Figure 1. Balanced *S. pneumoniae* D39 SpnIII locked allele inputs. Input colony forming unit (CFU) loads were standardised for experiments. A multiplicity of infection (MOI) of ~100:1 (CFU:A549) was used for adherence and invasion to A549 cells **(A)** and ~5:1 (CFU:Airway Cell) for adherence to human airway cells **(B)**. 1000 CFU per well were used in neutrophil killing and opsonophagocytic killing assays **(C)**. Approximately 50 000 CFU per well were used in the whole blood killing assay **(D)**.

Supplementary Figure 2

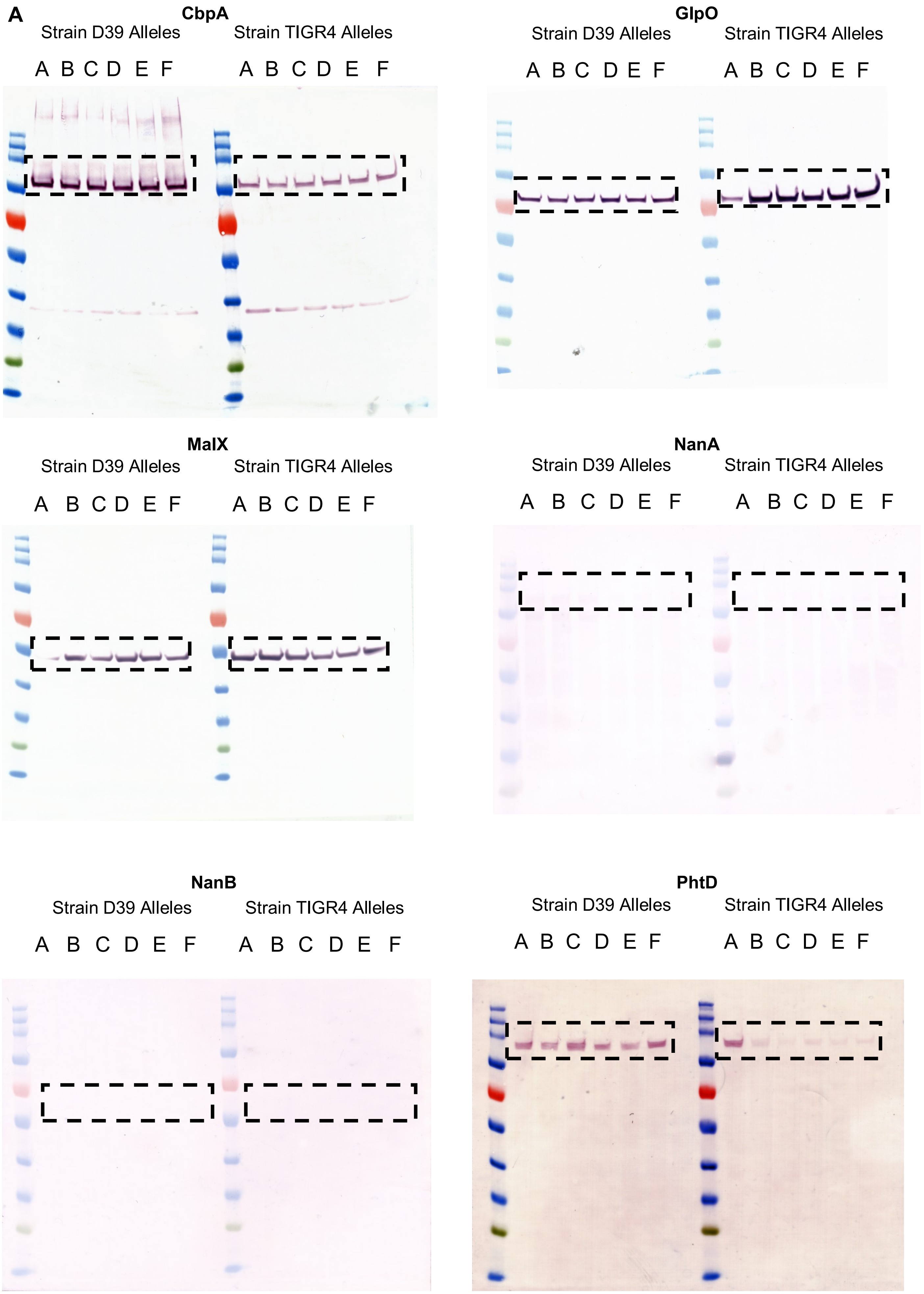

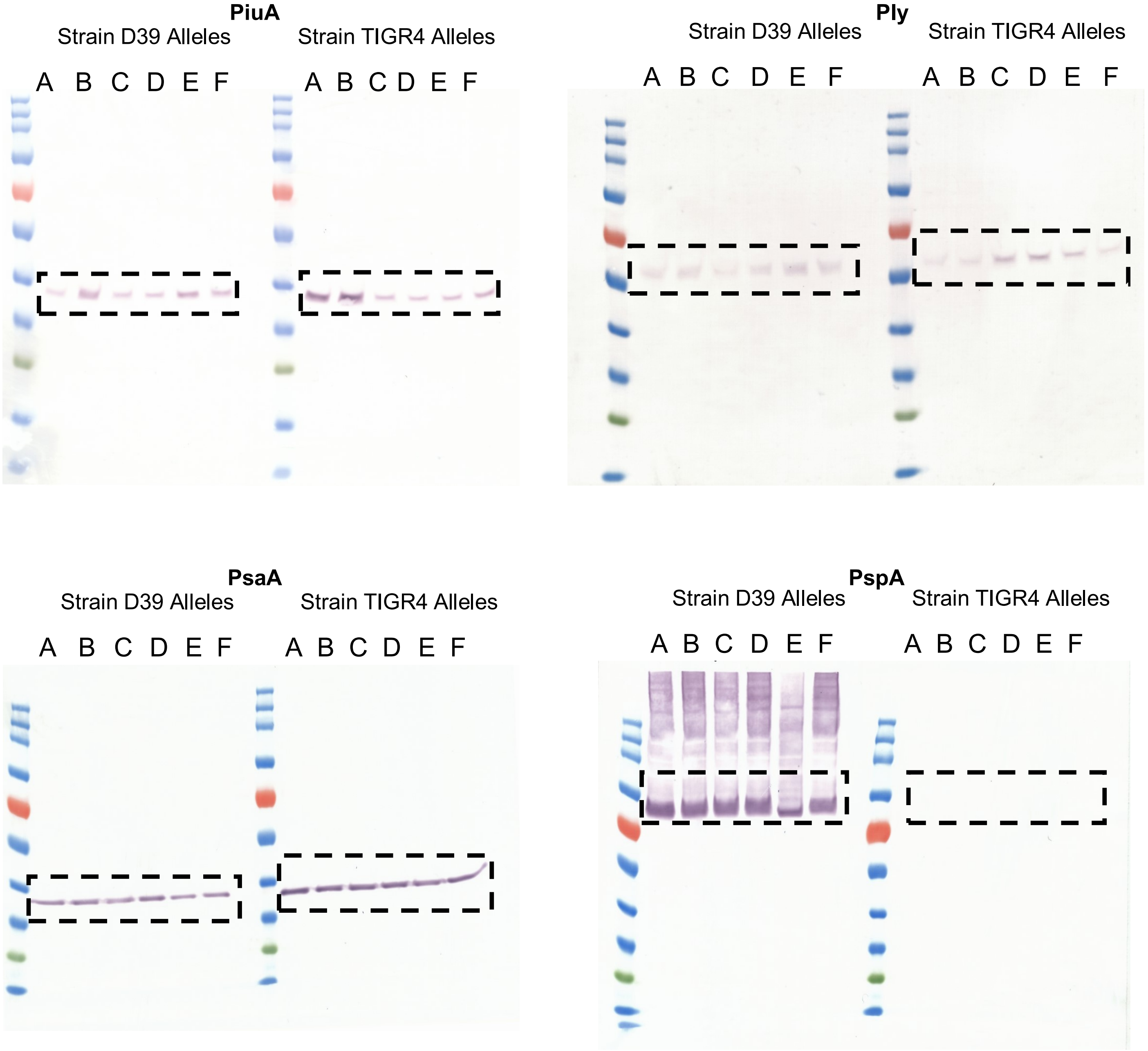

Supplementary Figure 2A. Full images of vaccine candidate Western Blots. Full scans of Western Blots using vaccine candidate anti-sera against *S. pneumoniae* D39 and TIRG4 locked SpnIII alleles. The outlined section of each Blot has been used in **Figure 3**.

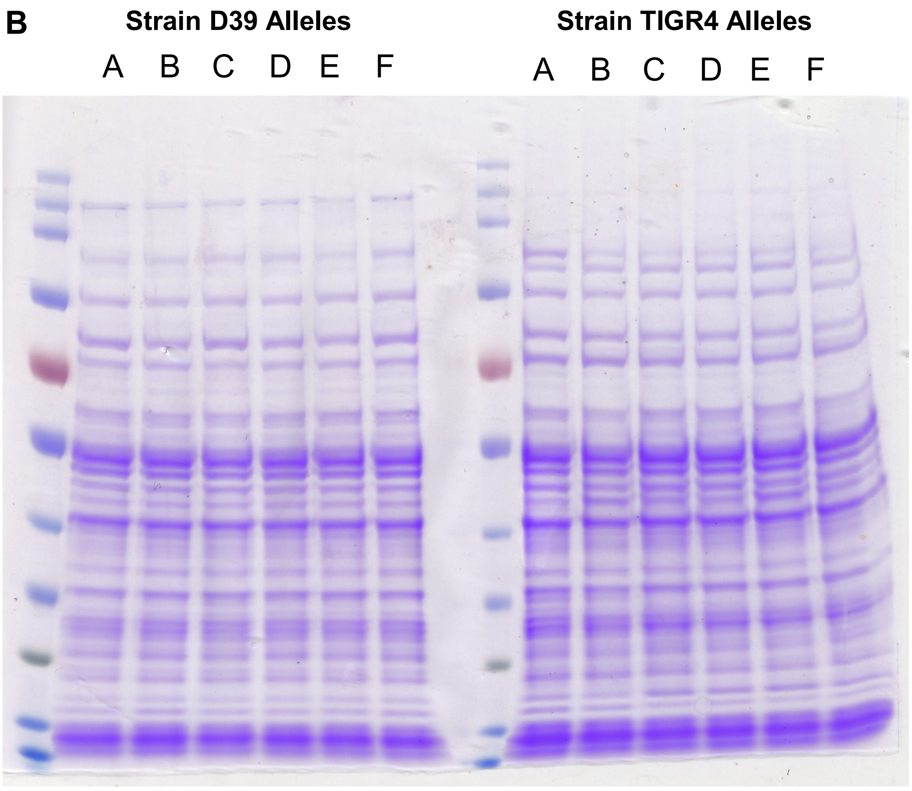

Supplementary Figure 2B. Balanced loading of locked allele lysates for Western Blots. D39 and TIGR4 locked allele cell lysate loads were standardised via Coomassie stain to ensure equal protein loading was used in vaccine candidate Western Blots used in **Figure 3** and **Supplementary Figure 2A**.

C

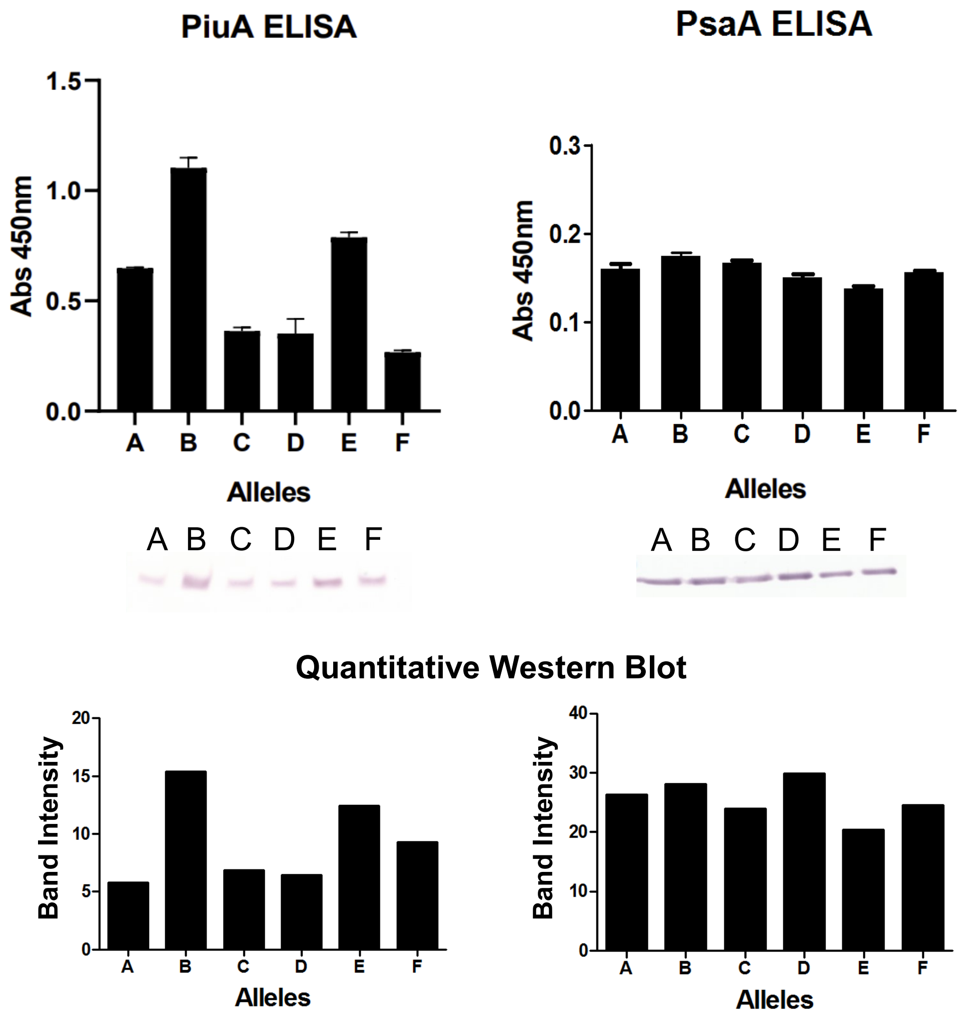

Fold Difference from Most Intense Band

PiuA Fold Difference	
B vs A	2.7
B vs C	2.3
B vs D	2.4
B vs E	1.2
B vs E	1.7

PsaA Fold Difference	
D vs A	1.1
D vs B	1.1
D vs C	1.2
D vs E	1.5
D vs F	1.2

Supplementary Figure 2C. PiuA and PsaA ELISA with comparative Western Blot and quantified Western Blot values. Western Blot banding intensity was quantified in ImageJ. Fold differences calculated from the quantified Western Blot values are provided, using the most intense band as a baseline for comparison.
